# Mental health of refugees following state-sponsored repatriation from Germany

**DOI:** 10.1186/1471-244X-8-88

**Published:** 2008-11-10

**Authors:** Ulrike von Lersner, Thomas Elbert, Frank Neuner

**Affiliations:** 1 Psychotrauma Research- and Outpatient Clinic for Refugees, University of Konstanz, Germany

## Abstract

**Background:**

In recent years, Voluntary Assisted Return Programmes (VARPs) have received increasing funding as a potential way of reducing the number of refugees in EU member states. A number of factors may affect the mental well-being of returnees. These include adjustment to the home country following return, difficult living conditions, and long-term effects resulting from the severe traumatic stress that had originally driven the affected out of their homes. Little is known about the extent to which these and other factors may promote or inhibit the willingness of refugees to return to their country of origin. The present pilot study investigated refugees who returned to their country of origin after having lived in exile in Germany for some 13 years.

**Methods:**

Forty-seven VARP participants were interviewed concerning their present living conditions, their views of their native country, and their attitudes towards a potential return prior to actually returning. 33 participants were interviewed nine months after returning to their country of origin. Mental health and well-being were assessed using the questionnaires Posttraumatic Stress Diagnostic Scale (PDS) and EUROHIS and the structured Mini International Neuropsychiatric Interview (M.I.N.I.).

Our objectives were to examine the mental health status of refugees returning to their home country following an extended period of exile. We also aimed to assess the circumstances under which people decided to return, the current living conditions in their home country, and retrospective returnee evaluations of their decision to accept assisted return.

**Results:**

Prior to returning to their home country, participants showed a prevalence rate of 53% for psychiatric disorders. After returning, this rate increased to a sizeable 88%. Substantial correlations were found between the living situation in Germany, the disposition to return, and mental health. For two thirds of the participants, the decision to return was not voluntary.

**Conclusion:**

Psychological strain among study participants was of a considerable magnitude. As a result of traumatic stress experienced during war and refuge, victims were vulnerable and not well equipped to cope with either post-migration stressors in exile or with a return to their country of origin. It is noteworthy that the majority returned under pressure from immigration authorities. Living conditions after return (such as housing, work, and health care) were poor and unstable. Participants also had great difficulty readapting to the cultural environment after having lived abroad for an average of 13 years. Current VARPs do not take these factors into account and are therefore not able to assist in a humanitarian reintegration of voluntary returnees.

## Background

Migration is a global phenomenon. An important driving power of migration is globalization and its integration of worldwide economic markets [1]. The majority of migrants, however, do not leave their home region for economic reasons. According to the latest report of the United Nations High Commissioner for Refugees, in 2006, approximately 33 million people worldwide were living in refugee-like situations. Only some 10% of these live in Europe [2]. These refugees seek protection from war, repression, and persecution. As a consequence of growing migratory movement, managing the integration or return of refugees has become a key issue for the governments of receiving countries. One measure which in recent years has received the support of the European Union (EU), including Germany, is the implementation of voluntary assisted return programmes (VARP). These state-sponsored programmes offer financial and practical assistance to refugees returning to their native land. They aim to promote sustainable and humanitarian reintegration in the country of origin. A wide range of federal agencies and non-governmental organisations are supported by a sizable budget in their efforts to facilitate assisted return. Entenmann and ZIRFcounselling provide an overview of return programmes in Germany and the EU [3,4].

In VARPs, emphasis has been placed on the 'voluntariness' of programme participants. According to Morrison, there is no single working definition of voluntariness which is generally accepted by all VARP agencies [5]. The UNHCR Handbook on Voluntary Repatriation defines voluntariness as the "absence of any physical, psychological, or material pressure. One of the most important elements in the verification of voluntariness is the legal status of the refugee in the country of asylum." [6]. The definition provided by the International Organisation for Migration (IOM) states "...that voluntariness exists when the migrants' free will is expressed at least through the absence of refusal to return, e.g., by not resisting boarding transportation or not otherwise manifesting disagreement" [7]. A number of empirical studies, cited in Black, Koser & Munk, argue that voluntariness generally arises in connection with the fact that rejected asylum seekers are not assisted by the receiving country and are faced with the reality of deportation; a fact which in many cases leads to the decision to 'voluntarily' return [8].

Similarly, there is no commonly accepted definition of the term 'sustainable humanitarian reintegration'. Black et al. state that "the simplest measure of sustainability of return would be whether those who do return subsequently re-emigrate" [8]. The UN Mission in Kosovo goes further in stressing that "return can only be considered sustainable where returnees are able to gain access to rights to services, shelter, and freedom of movement" [9].

Dahinden defines different aspects of reintegration and distinguishes between structural and cultural reintegration [10]. While the former includes access to labour, education, social life, and so forth, the latter pertains to cultural norms and values. A third aspect comprises personal identification with a specific culture, which in returnees can tend toward either the receiving country or the country of origin. In order to attain sustainability, all three aspects of reintegration must be realised.

In the absence of a general definition, there are no defined standards on how to achieve sustainability, that is, there are no guidelines explaining which steps must be taken by return programmes. Consequently, each player involved in VARP currently has its own agenda, as a result of which the comparison and evaluation of return programmes is severely complicated.

To date, only very little research has examined the sustainability of return programmes. As shown above, the assessment of sustainable reintegration is complicated by the lack of a valid benchmark. Black and Gent assume that programme evaluation is avoided in order to conceal the extent to which return policy has failed or proven unsustainable [11].

### Motivation to return

The concept of 'voluntary return' as one possible solution to the refugee question is not new. Nevertheless, there is currently no scientifically sound research on VARPs.

Early sociological and anthropological research in this field focused on the motives and expectations of voluntary returnees upon deciding to return to their country of origin. It is important to note that this work was based on samples of migrant labourers. Many of the findings can, however, also be applied to refugees. An important approach in this context is the model of 'push' and 'pull' factors. Pull factors attract the potential returnee away from the receiving country and back towards their country of origin. Some of these pull factors include family ties, homesickness, and a sense of national loyalty.

These factors are coupled with push factors which make a prolonged stay in the receiving country unattractive and pressure – or push – the potential returnee to leave. Such factors include insufficient monetary funds, insecure visa or residential status, discrimination, and even the inability to adjust to weather conditions in the receiving country [10,12].

Several studies have demonstrated that pull factors play a greater role in the decision to return and that non-economic factors weigh more heavily than economic factors [8,13,14]. These findings have also been replicated in refugee populations [5]. A study by Al-Ali et al. revealed that economic and social problems in the country of origin as well as a desire to first complete their children's education represent important motives in deciding not to return [15]. Similar conclusions were drawn by von Lersner et al [16,17].

A further classification of return motives has been proposed by Gmelch [13]. He allocates the motives for return into three categories: 1) familial-personal reasons, 2) economic-occupational reasons and 3) social-patriotic reasons [13].

**Figure 1:**
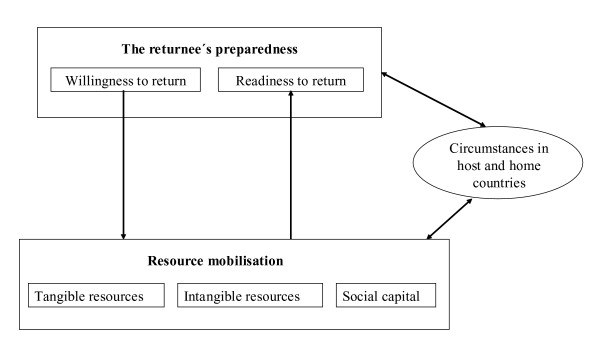
Resource mobilisation and the returnee's preparedness.

A model developed by Cassarino approaches voluntary return from a different angle, incorporating those factors which are relevant for success, that is, for the sustainability of return [18]. According to this model, 'preparedness' of returnees is the crucial prerequisite for sustainability. Being 'prepared' assumes that a refugee expresses the willingness, that is, the voluntariness to return. The refugee then begins to mobilise all available tangible and intangible resources necessary in the preparation for return and a new start in the country of origin. In addition to a social network (which can also include VARPs), a sufficient amount of time is required to complete resource mobilisation and achieve 'readiness'. According to Cassarino, return can only be successful (in terms of sustainability and reintegration) if these conditions are fulfilled. Figure 1 illustrates interactions between aforementioned factors and between these factors and circumstances in the receiving country and the country of origin [18].

### Mental health of refugees and asylum seekers

Before being forced to leave their home country, refugees and asylum seekers are often victims of organised violence, including civil war and torture. The flight itself is often very stressful and extends over long periods of time. Upon arrival in receiving countries, refugees have thus frequently been subject to a number of traumatic experiences and demonstrate a high level of distress. People who experience considerable emotional distress and traumatic events commonly develop posttraumatic stress disorder (PTSD). The percentage of trauma-related psychiatric disorders and level of functional impairment found varies according to the number of traumatic stressors experienced by the sample under investigation as well as the respective socio-political context [19-22]. Considerable variance in the prevalence of PTSD is thus to be expected when comparing refugees from different countries [21,23]. For refugees from the former Yugoslavia who are now living in exile, reported rates of PTSD range from 30% to 60% [16,24-26]. A PTSD rate of 17% has been reported in the population of Kosovo and a rate of 18% among family physicians in Bosnia [27,28]. A study by Hunt and Gakenyi suggests that emotional distress and psychiatric disorders are more frequently observed in refugees who have left their own country as compared with individuals who remain in their home region or who are internally displaced [29]. As shown by Marshall et al., this difference may continue to persist even after 20 years in exile [30]. The authors explain the higher level of psychiatric disorders as being due to additional stressors caused by flight and life in exile. The latter are referred to as 'post-migration stressors' and include difficulties in becoming integrated in or learning the language of the host country, an unstable visa status, social isolation, and discrimination [31,32]. Steel et al. examined the influence of the living conditions of refugees on the development and perpetuation of PTSD. They found that post-migration factors (such as integration difficulties, loss of contact with one's cultural roots) explained 14% of the variance in PTSD-pathology and pre-migration factors 20% [32]. A significant correlation between stressors in exile (such as low activity levels or social isolation) and symptoms of depression was also found in Bosnian refugees [21]. In a longitudinal study, Lehmann and Ruf demonstrated that while the attainment of permanent residential status led to a decrease in symptoms of depression, it had no influence on posttraumatic stress disorder [33,34]. Sundquist et al. examined the subjective quality of life and general health of female Bosnian refugees in Sweden as compared with native Swedish women [35]. For those individuals who work with refugees, it will come as no surprise that the group of Bosnian women attained significantly inferior results to those obtained by controls in both categories.

### Mental health of returnees

It is only in recent years that the mental health of returnees has gained attention. Roth et al. interviewed refugees from Kosovo shortly after their arrival in Sweden as well as 3, 6, and 18 months later [36]. Upon arrival, 37% of the refugees were diagnosed with PTSD. 18 months later, 52% of those who had returned to Kosovo and 87% of those who had remained in Sweden were diagnosed with PTSD. The authors suggest that additional post-migration distress experienced in exile was responsible for these results [37,38]. In a similar study, Toscani et al. examined the living conditions of those returning from Switzerland to Kosovo and found a PTSD rate of 25%. Sixty-five percent of the Kosovo returnees were living in extreme poverty and suffered from general poor health [39]. In this study, a negative correlation was found between the length of returnees' time in exile and their mental health following return. Unfortunately, no control group was included in this study. Sundquist et al. conducted a longitudinal study with Chilean and Uruguayan refugees in exile in Sweden [40]. In contrast to the studies presented above, those who returned home suffered more in terms of mental health and were less integrated in the country of origin than those who remained in Sweden. Level of mental stress, discrimination, and insecurity in everyday life were also higher among returnees. The contradictory results presented here reflect the lack of clear and scientifically founded information on the mental health of refugees in the process of returning to their home country.

### Project context

Thus far, no scientific data on the sustainability of assisted voluntary return programmes in Europe have been published. There is further no information available on the impact of the return process on the mental health of the persons concerned. This information is crucial in helping to develop guidelines for the successful social reintegration of returnees in their home country.

Against this backdrop, we conducted a longitudinal survey which was designed to analyse the phenomenon of 'voluntary return' from a psychological perspective. To this end, we investigated the present living conditions, mental health, quality of life, and motives for or against voluntary return in a sample of refugees in Germany. In a first stage, the motives of refugees from the former Yugoslavia who did not want to return to their home country and who preferred to remain in the receiving country (in this case Germany) were subject to investigation. Results are presented in von Lersner et al. [16,17].

In the present paper, we examined the living conditions, mental health, and subjective quality of life of returnees nine months after returning to their home country and compared their answers to data collected while still in exile. We strongly hope that the findings of the study will help to improve understanding of the situation in which returnees find themselves and in turn foster more reliable, applicable, and effective services in helping to reduce the suffering of those returning to their home country following a period of exile.

## Methods

### Experimental design

**Figure 2:**
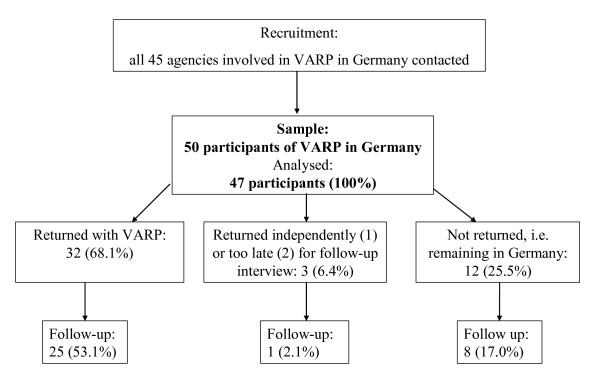
Progression of participants through the study.

Participants were recruited by means of advertisements posted in refugee centres, language schools, and doctors' surgeries (see Figure 2). In addition, all organisations in Germany involved in the management of voluntary return were contacted. For the first interview (pre-tests before returning to home country), participants were recruited and interviewed between June 2005 and March 2007. In recruiting the study group (returnees), a total of 45 organisations were contacted, 10 of which referred clients to us. The majority of refugees in our sample (25 participants) returned with the organisation 'AWO Heimatgarten' [41]. Seven organisations refused to cooperate, citing political reasons for their refusal. Other organisations made referrals which were too close to the scheduled return of their clients, leaving insufficient time for interviews to be conducted. The remaining organisations did not have any clients who met our inclusion criteria. Agencies generally reported a lack of clients to be a consequence of new legislation in 2006 regarding the right of residence for refugees who have been living in Germany for longer periods of time (resolution of the Conference of Ministers of the Interior, IMK) [42]. Participants were located in all regions of Germany and were interviewed in their homes or at local refugee centres.

For the second interview (follow-up), participants from the first interview were re-contacted. Those who had returned to their home countries were interviewed nine months after returning. Thirty-two of the 47 participants from the first interview had returned. Twelve participants who had intended to return in the first interview had dropped out of the return programme and remained in Germany. These participants were also interviewed for a second time nine months after the first interview. Reasons for dropping out of the programme included attainment of a permanent residence status in Germany and the extension of a current permit for a further two years; participants thus sought permission to remain in Germany parallel to their involvement in the return process. Further reasons were insufficient cooperation in preparing to return or dissatisfaction with the programme's services, in which case people returned on an individual basis. Eight of the 12 participants who had dropped out of the programme were willing to take part in the follow-up interview.

Follow-up interviews were conducted with 25 of those participants who had returned. Of the remaining seven participants who returned without being interviewed a second time, one had died of cancer shortly after return, one had illegally returned to Germany, one could not be contacted at the given address, and five returned to regions in Iraq which were too dangerous to travel to for the follow-up interviews. Table 1 provides an overview of the study sample.

**Table 1:** Demographic characteristics

		**Pre-Test**	**Post-Test**		**Statistics**	
		***Total *** ***(n = 47)***	***Returned with VARP *** ***(n = 25)***	***Not returned *** ***(n = 8)***		
**Sex *N *(%)**	Male	25 (53.2)	12 (48.0)	4 (50.0)	Fisher's Exact	*p *= 1.0
	Female	22 (46.8)	13 (52.0)	4 (50.0)		
**Age in years *M (SD*)**		48.7 (17.2)	53.5 (17.4)	46.4 (11.7)	Fisher's Exact	*p *= .4
**Older than 60 years (%)**		9 (19.1)	6 (24.0)	0 (0)		
**Marital status (%)**	Single	7 (14.9)	2 (8.0)	- (-)	*Chi*^2 ^(3) = 5.1	*p *= .2
	Married	29 (61.7)	14 (56.0)	8 (-)		
	Divorced	6 (12.8)	4 (16.0)	- (-)		
	Widowed	5 (10.6)	5 (20.0)	- (-)		
**Country of origin**	Bosnia	9 (19.1)	6 (24.0)	2 (25.0)	*Chi*^2 ^(3) = 4.2	*p *= .1
	Serbia	16 (34.0)	11 (44.0)	1 (12.5)		
	Kosovo	12 (25.5)	5 (20.0)	5 (62.5)		
	Turkey	5 (10.6)	3 (12.0)	- (-)		
	Iraq	5 (10.6)	- (-)			
**Number of children *M *(*SD)***		4.2 (3.2)	4.6 (2.7)	5.14 (4.1)	*U *= 73.0	*p *= .9
**School aged children *N *(%)**	Total	19 (40.4)	12 (48.0)	4 (50.0)	*Chi*^2 ^(2) = 3.6	*p *= .2
	In school	19 (100.0)	4 (33.0)	4 (100.0)		
	Not in school	- (-)	6 (50.0)	- (-)		
**Duration of stay in Germany in years *M (SD)***		13.1 (4.2)	13.0 (3.5)	13.4 (.9)	*t *(17.7) = -.5	*p *= .6
	Minimum	3	3	12		
	Maximum	18	17	14		
**Education in years *M (SD)***		7.7 (4.9)	6.5 (5.1)	10.2 (.5)	*t *(4.2) = -.7	*p *= .5

The majority of the interviews were conducted in co-operation with trained interpreters, allowing the participants to express themselves in their own language. All participants completed written consent forms, approved but the Konstanz University Ethical Review Board and were assured that interviews were confidential. Interviews lasted approximately two hours. Participants who experienced distress following the interview were referred to local health professionals.

### Participants

Participants were 47 refugees from the former Yugoslavia, Iraq, and Turkey living in Germany who had decided to voluntarily return to their countries of origin. Participants were interviewed by trained interviewers from the Outpatient Clinic for Refugees at the University of Konstanz. These three countries were selected on account of the fact that they had the highest number of voluntary returnees in 2006 [43]. Furthermore, the former Yugoslavia was the destination of the largest number of voluntary refugee returns from European countries in the last decade, and has had an important influence on the way in which governments, international organisations, and non governmental organisations (NGOs) approach voluntary return [8].

Inclusion criterion was the provision of a written informed consent form for participation in an assisted voluntary return programme. Participants were between the ages of 19 and 90 years, with an average age of 49 years. Forty-seven percent were female; the average length of education was 7.7 years. The average duration of stay in Germany was 13.1 years.

Demographic characteristics are presented in Table 1 in the form of descriptive statistics for the total sample as well as the two groups of returnees and non-returnees, separately. There were no significant differences between returnees and those who remained in Germany.

Due to emotional distress caused by the topics discussed within the interviews or severe psychological disability, not all participants completed the entire assessment. In four cases, only demographic characteristics and information from medical records were included in the database.

### Data analysis

Data were coded and analysed using the SPSS package and demographic data examined using descriptive statistics [44].

Due to the fact that some participants refused -or were not able- to answer certain questions, the sample size varies across interview sections. For this reason, sample size is reported for each analysis.

### Outcome measures

#### Demographics and return

This questionnaire was designed to collect information on the living conditions of the participants. It includes questions regarding origin, ethnicity, religion, age, sex, marital status, level of education, employment, and clinical history. Further questions target the reasons for and circumstances of the participant's flight, duration of stay in Germany, current living situation in Germany, and attitude towards a voluntary return. At follow-up, questions concerning living situation after return and attitude towards the decision to return as well as the return process were included. A further section of the questionnaire covers participants' motives in opting for or against a voluntary return to their country of origin. In line with Gmelch, these motives were divided into three categories: 'familial-personal reasons', 'economic-occupational reasons', and 'social-patriotic reasons' [13].

#### Posttraumatic stress

The Posttraumatic Stress Diagnostic Scale (PDS) was used to assess symptoms of posttraumatic stress [45,46]. The scale is a self-report questionnaire which is designed to aid the detection and diagnosis of PTSD. It consists of a traumatic event scale and a symptom scale. The symptom scale comprises 17 items and the subscales 'intrusions', 'avoidance', and 'hyperarousal'. It is closely modelled on DSM-IV criteria for PTSD and may be repeatedly administered in order to help monitor symptom change [47]. Participants were asked to indicate the frequency of each symptom over the past four weeks using a four-point Likert scale which ranged from 0 (not at all or only once) to 3 (five or more times per week/almost always). In the present study, the PDS was used in the form of an interview.

#### Mental health

Psychological functioning was measured using the German version of the Mini International Neuropsychiatric Interview (M.I.N.I.), Version 5.0.0 [48,49]. The M.I.N.I. is a short structured diagnostic interview for DSM-IV and ICD-10 psychiatric disorders. Validation studies have demonstrated good validity and reliability in making diagnoses in less time than conventional structured interviews such as the Structured Clinical Interview for DSM-IV-Patient Edition or the Composite International Diagnostic Interview [49-51]. In the present study, Sections I (PTSD), L (psychotic disorders) and P (antisocial personality disorder) were not included in the interview.

#### Quality of life

The EUROHIS-QOL eight-item index is a subjective measure of quality of life, derived from the WHOQOL-100 and the WHOQOL-BREF [52,53]. The overall QoL score is formed by summating the scores of the eight items. Higher scores indicate better QoL. Conceptually, the four domains measured (psychological, physical, social, and environmental QoL) are each represented by two items. Each item is answered on a five-point Likert scale ranging for instance from 1 (not at all) to 5 (completely). A study by Schmidt et al. revealed good reliability and validity of the measure across a range of countries [54].

## Results

### Living conditions after return

Sixty-four percent of returnees (16 participants) returned to the place where they had lived before fleeing. Nine months after return, 36% (nine participants) lived with relatives and 64% (16 participants) lived separately. For almost all returnees, housing conditions were very basic and crowded, and several houses were of a makeshift nature.

Eighty percent (20 participants) returned together with family members. Twenty percent (five participants) reported difficulties with state authorities following their return due to either political activities prior to fleeing or to their minority status.

Eight percent (two participants) had a regular income. The remaining participants reported irregular income from belongings which they sold in order to finance their daily living or from short-term labour. Fifty-six percent (14 participants) received financial support from a return programme (amounts ranged from 50 euros per month to 550 euros for periods of between five months and two years). The majority (80%) lived from sporadic donations from friends and family members who lived in the country of origin or who had remained in Germany. On the whole, the economic situation of returnees can be characterised as difficult.

As reported in von Lersner et al., the future and the education of their children was the main argument against voluntary return among returnees [16]. Before returning, 76% (19 participants) had school-aged children, all of whom attended school in Germany. After returning, 48% (12 participants) had children of school age. Of those, 17 percent (two participants) returned without their children, 33% (four participants) sent their children to school in the country of origin, and 50% (six participants) did not. The reasons for not sending their children to school included a lack of financial resources, minority status, language difficulties of the children, and refusal of the children to adapt to the 'new' school system.

Returnees had lived in Germany for an average of 13.1 years. Before returning, 46% (12 participants) stated that Germany rather than their country of origin was the place in which they felt at home. Nine months after returning, 50% (13 participants) were of the same view. The longer refugees had lived in Germany, the less they felt at home in their country of origin. This was the case both before (*r *= -.50, *p *= .04) and after returning (*r *= -.45, *p *= .02) to their homeland.

In response to the question concerning whether they felt integrated in their social environment following their return, 62% (16 participants) answered in the negative. Twenty-seven percent (seven participants) stated that they only had contact with family members. Nineteen percent (five participants) reported that they were also in regular contact with other returnees, but not with other people in their environment. A further reason for feelings of isolation was the dismissive attitude of those who had remained in the country of origin towards returnees. Sixty-two percent (16 participants) reported that those who had remained in the country expected them to be rich. Twenty-three percent (six participants) reported having been put under pressure and/or having had money and other goods stolen by neighbours shortly after their return. Forty-four percent (11 participants) reported feeling safe in their environment, while 36% (nine participants) reported having been discriminated against after return either for being a returnee (20%, five participants) or for their ethnicity (16%, four participants).

When asked to judge their satisfaction with having returned, 32% (eight participants) reported that they were glad to have returned, while 52% (13 participants) were not content. 68% (17 participants) stated they would prefer to re-emigrate to Germany. Reasons for wanting to re-emigrate were as follows:

"People here are completely different from me. I cannot relate to them." (female, 50)

"I feel like a burden to my son who had to sell everything for my medical treatment." (male, 39)

"Even though I myself prefer to live here in Serbia, I would like to return for the sake of my children." (female, 38)

"In Germany, the legal system works and human rights count." (male, 50)

"In Germany I felt safe, here I feel scared because I am a Bosnian among Serbs." (male, 40)

"I would like to return to Germany because of the good health care system."(male, 40)

"Our friends are in Germany, we were integrated. It was our home!"(male, 46)

It should be noted that prior to their return, 52% (13 participants) of those who finally returned reported that their decision to return was not voluntary. From the entire group in the pre-test (*n *= 47), 58% (27 participants) reported that their return was involuntary and highly influenced by government authorities, and was therefore seen as an alternative to forced return. Those participants who perceived their country of origin as 'home' before returning demonstrated a higher willingness to return (*r *= .81, *p *= .00). Statistical analysis revealed a negative correlation between duration of stay in Germany and voluntariness of return (*r *= -.50, *p *= .02).

We further examined motives for flight, return, and re-emigration over time. The initial motives for leaving the country of origin comprised political reasons related to war and ethnic repression, for example orders to serve in the army (28%, seven participants); political persecution (16%, four participants); ethnic discrimination (16%, four participants); and living in a war zone (40%, 10 participants). Motives for return were a lack of self-determination in Germany (8%, two participants); the desire to die in the country of origin (28%, seven participants); and the avoidance of forced return (64%, 16 participants). When asked about potential considerations for re-emigration, participants reported motives such as a lack of adequate safety (16%, four participants), a lack of adequate health care (24%, six participants), and poor living conditions, such as unemployment and poverty (60%, 15 participants).

A significant positive correlation was found between voluntariness of the decision to return (measured before return) and age (*r *= .54, *p *= .01). The main motive for a voluntary return for participants above the age of 60 was the desire to die in their home country and to see their family members again before dying. Interestingly, while older participants seem to voluntarily return more often, they also seem to miss Germany more after returning (*r *= .43, *p *= .04).

### Mental health

In this section, we will first present results of the pre-tests (*n *= 47) followed by results of the follow-up (*n *= 33). Table 2 provides an overview of both sets of results. Results are presented separately for those who returned with VARP (*n *= 25) and those who remained in Germany (*n *= 8).

**Table 2:** Mental health in returnees before and 9 months after their return (%)

***Psychiatric disorder***	***Total pre *** ***(n = 47)***	***Returned pre *** ***(n = 25)***	***Returned post *** ***(n = 25)***	***Not returned pre *** ***(n = 8)***	***Not returned post *** ***(n = 8)***
**At least one DSM-IV diagnosis**	25 (53.2)	14 (56.0)	22 (88.0)	6 (80.0)	7 (87.5)
**PTSD**	14 (29.8)	9 (36.0)	14 (56.0)	3 (37.5)	3 (37.5)
**Depression**	15 (31.9)	7 (28.0)	16 (64.0)	3 (37.5)	5 (72.5)
**Manic Episode**	- (-)	- (-)	1 (4.0)	- (-)	- (-)
**Dysthymia**	5 (10.6)	2 (8.0)	1 (4.0)	1 (12.5)	1 (12.5)
**Suicidal Tendencies**	14 (29.8)	9 (36.0)	11 (44.0)	2 (25.0)	2 (25.0)
**Psychotic Disorder**	4 (8.5)	2 (8.0)	2 (8.0)	1 (12.5)	1 (12.5)
**Agoraphobia**	4 (8.5)	3 (12.0)	- (-)	1 (12.5)	- (-)
**Panic Disorder**	3 (6.4)	2 (8.0)	1 (4.0)	1 (12.5)	- (-)
**Social Phobia**	- (-)	- (-)	1 (4.0)	- (-)	- (-)
**Obsessive-Compulsive Disorder**	- (-)	- (-)	- (-)	- (-)	- (-)
**General Anxiety Disorder**	2 (4.3)	1 (4.0)	2 (8.0)	1 (12.5)	1 (12.5)
**Eating Disorder**	- (-)	- (-)	- (-)	- (-)	- (-)
**Substance Abuse/Dependence**	1 (2.1)	- (-)	- (-)	- (-)	- (-)

Undergoing psychological treatment	18 (38.3)	13 (52.0)	4 (16.0)	3 (37.5)	3 (37.5)

#### Pre-test

Prior to return, 53% (25 returnees) were diagnosed as meeting the criteria for at least one psychiatric disorder according to DSM-IV criteria. As shown in Table 2, the most frequently detected disorder was depression, followed by PTSD. High rates of suicidal tendency were also found. Thirty-eight percent of the returnees reported having consulted a psychotherapist and/or psychiatrist.

Thirty percent of returnees were diagnosed with PTSD prior to their return. But also those returnees who were not diagnosed with PTSD, 43 participants reported traumatic events. Among the traumatic events reported by all returnees, experiences relating to war and violence occurred most frequently (30% war-related events, 28% related to the witnessing of a violent attack, 26% experiences of violence against one's own person). Among the 25 participants who later returned with VARP, 56% (14 participants) presented at least one psychiatric disorder according to DSM-IV criteria. As shown in Table 2, the most frequently occurring disorders in this subgroup were also PTSD and affective disorders, followed by anxiety disorders.

#### Follow-up

**Figure 3:**
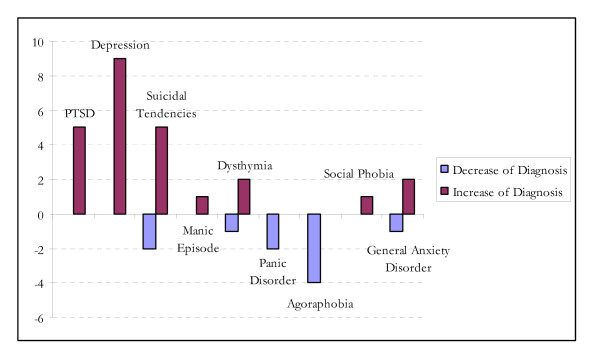
Increase and decrease in psychiatric disorders: comparison before and after return.

Following return to their home country, 88% of returnees (22 of 25 participants) were diagnosed with at least one psychiatric disorder. The data reveal that PTSD and depression were not only the most frequently occurring disorders in this group, but also the disorders with the greatest increase in prevalence following return (see Figure 3). Thirty-six percent (9 participants) developed depressive symptoms and 20% (5 participants) developed PTSD who had not been diagnosed with these disorders in the pre-test. Parallel to the development of these disorders, an increased intensity of suicidal tendency was found, with three participants reporting levels which had increased from low to high. Anxiety disorders such as panic disorder and agoraphobia were no longer present in two participants at follow-up.

At the level of the individual, 15 participants showed a decline in mental health, 2 participants improved, and 9 participants showed no change.

While prior to returning 52% (13 participants) had been in psychological or psychiatric treatment in Germany, only 16% (4 participants) consulted a psychiatrist on a regular basis or were treated on a psychiatric ward back in their home country.

In the group of participants who dropped out of VARP and remained in Germany, psychological strain was already at a high level in the pre-test (see Table 2). At follow-up, the frequency of psychiatric disorders did not change with the exception of two additional participants who developed depression.

In the group of those who returned, statistical analyses revealed a significant increase in the frequency of psychiatric disorders in general (*p *= .008) and depression in particular *(p *= .008) following return. A trend toward significance was found for PTSD (*p *= .06).

A closer examination of those five participants who developed PTSD after return reveals that in three cases, traumatic events experienced prior to the flight to Germany (war-related events) were crucial in triggering PTSD at follow-up. The affected individuals reported intrusions and nightmares which were related to these traumatic events. In the other two cases, traumatic events experienced after returning to the country of origin were responsible for the development of PTSD.

Statistical analyses revealed a significant negative correlation between attitudes towards the country of origin as being 'home' and both depression (*r *= -.59, *p *= .01) and suicidal tendencies (*r *= -.49, *p *= .02).

### Subjective quality of life

In the group of participants who returned to their country of origin (*n *= 25), average subjective quality of life (QoL) prior to return was *M *= 3.2 (*SD *= .7) and *M *= 2.3 (*SD *= 1.1) following return. This difference in QoL before and after return proved significant (*t *(18) = 4.0, *p *< .01). In the group of participants who did not return and remained in Germany, QoL significantly increased from pre-test (*m *= 2.4, *SD *= .6) to follow-up (*m *= 2.8, *SD *= .4) (*t *(2) = -5.0, *p *< .05).

No significant differences in QoL were found between participants with at least one psychiatric disorder and. Also no significant differences were found between those participants who actually returned and those who remained in Germany neither in the pre-test nor in the follow-up.

## Discussion

### Mental health and quality of life after return

The results of the study presented in this paper show that psychological distress among participants of VARP is high prior to returning to their country of origin. As also demonstrated in earlier studies the most frequently diagnosed disorders in refugee populations are depression and PTSD [22,25,37]. Rates of suicidal tendency in this population also increased at follow-up.

Nine months after returning to their home country, the frequency of psychiatric disorders further increases, with depression and PTSD increasing the most. Increases in suicidal tendency at follow-up correlate with these disorders.

Before returning, half of the group of returnees had consulted a therapist on a regular basis. Multiple reasons account for this change. Upon returning, only a small number of returnees received psychiatric treatment and none consulted a psychotherapist. Besides the fact that most returnees are unaware of the severity of their psychiatric status, many regions to which people return provide little opportunity for psychiatric or psychotherapeutic treatment. Moreover, due to a lack of financial resources, returnees are generally unable to pay for psychological treatment which they received for free in Germany. In light of the difficult economic situation of households examined and the fact that basic needs often cannot be met, psychotherapy becomes an unaffordable luxury. A further reason for the observed decline in treatment is the image of psychology in the countries of the Former Yugoslavia. Here, the concept of mental health itself is relatively new and people seeking treatment are easily stigmatized, as a result of which many do not take advantage of such services.

After return, our data show an increase in symptoms of PTSD. In two of the five persons concerned, PTSD was caused by traumatic events which occurred after return. In the remaining three participants, posttraumatic symptoms (such as intrusions and nightmares) were related to events which had been experienced before fleeing to Germany. In their content, these intrusions and nightmares were related to war and ethnic repression. In the pre-test, these participants had reported single symptoms but had not met the diagnostic criteria for PTSD. These cases might be classified as delayed-onset PTSD. Delayed-onset PTSD is a phenomenon in which individuals who have experienced traumatic events do not report PTSD symptoms until several years later [47,55]. It would seem plausible that a fear network underlying PTSD had been formed during war experiences and was largely inhibited during exile [22,56]. Confrontation with the traumatising environment and other stressors related to the return might have caused a decomposition of inhibitory mechanisms.

Apart from PTSD, other psychiatric disorders and in particular depression were more frequently diagnosed following return. Due to the small sample size, we were not able to identify clear predictors for this strong increase. Nevertheless, a number of potentially influential factors may be hypothesized. These may on the one hand comprise objective living conditions after return which are characterised by a lack of resources and unmet basic needs. As described, participants returned to living conditions which were very difficult and highly unstable. Some returnees lived from the money that they received from the VARP; money which will run out at the latest after two years. When discussing the living situation of returnees, we must, however, also take the living conditions of the general population in the country of origin into account. High rates of unemployment, bad housing conditions, and low income generally prevail in post-conflict countries such as those to which participants returned. In contrast to those who did not leave their home country during the war, however, returnees were confronted with additional stressors during flight and exile [29]. These additional factors may have increased their vulnerability to new stressors and unstable situations. Having lived abroad for an extended period of time, these returnees have also lost access to social networks which – as shown in the model by Cassarino – are essential in rebuilding a life after return [18]. Unfortunately, returnees are often discriminated against and/or isolated by those who did not leave the country, thus hampering reintegration. As shown in our study, participants stated that they had been confronted with discrimination upon returning. Almost the entire group reported having been faced with demands for money (or having been robbed) as people expected them to be wealthy.

Perceptions of the situation in the country of origin also distinguish returnees from people who did not leave the country during the war period. Such subjective reasons may ultimately be more relevant for the development of psychiatric disorders.

This is reflected in the significant decline in subjective quality of life (QoL) reported after return. According to Franz, this measure not only describes clients' perception of their quality of life but also the degree of adaptation to their living conditions [57]. Returnees thus not only report a lower quality of life after return on account of objective circumstances but also based on a comparison of their current situation to their living conditions back in Germany. In this sense, a crucial problem in terms of re-integration is the lack of accordance between returnees' expectations and the society with which he or she is confronted after return. Black et al. state that "...return is in any case very unlikely to be to the 'status quo ex ante'. Yet, in practice, the experience of return may be more, rather than less problematic than the experience of exile" [8]. As can be concluded from the statements of returnees ("Everything is different.", "I cannot relate to the mentality anymore.") and the high rates of participants who reported feeling more at 'home' in Germany than in their country of origin, people do not identify with their environment after return. These results also indicate that the notion of a fixed and 'clear' home is particularly problematic. Black and Gent suggest that refugees can feel more at 'home' in the country of asylum, in particular if they have lived there for a long time – as was the case for participants in our sample – or if they are likely to be denied economic or social opportunities in their country of origin [11].

In this context, we observed a phenomenon which has been described in the literature as 'dependency syndrome' [58]. This phenomenon describes the growing helplessness, apathy, and lack of self-dependent actions in refugees who live with the refugee status in Germany for an extended period of time. Participants in our study lived with an unstable visa situation for an average period of 10 years, were not allowed to work, could not afford language schools, and basically had no access to German society with the exception of social workers and immigration authorities. These refugees were forced to live from the welfare system and to remain idle. We hypothesize that with time, they became more and more dependent, apathetic, and incapable of independently organising their life up to the point that many refugees developed depression, as seen in the high diagnosis rates. While this observation can not be generalized to the entire group of refugees in Germany, it most likely applies to those who spent a long period of time in exile and who did not make use of earlier opportunities to return [59,60]. Our findings show that many returnees withdraw from their environment after return and simply avoid dealing with the new reality with which they are faced. They do not leave the house, do not send their children to school, watch German TV, and instead continue demanding that they be allowed to return to Germany. With reference to Oberg, Dahinden refers to this phenomenon as a 'reverse cultural shock', pointing out the parallels to the 'cultural shock' which people can go through when arriving in a different country [10,61]. In this sense, return "may not be a '*re*-' anything but the beginning of a new cycle" [8].

In our study, we also examined the motives for migration among study participants over time. While all participants fled from their country of origin for war-related reasons, personal and legal reasons were decisive for their return.

In contrast to model predictions, most returnees did not report pull factors in the country of origin as the key motive for their return, stating instead the fear of a deportation as a push factor. The most important argument cited against return was a desire to educate children and the conviction that the education system in Germany was better than that in the country of origin.

These findings are congruent with other studies which have found that "family and life cycle factors might be more important for returnees than for initial emigration" [8]. These results do not necessarily contradict the model of push-pull factors, since this model implies a voluntary decision to return which according to the statements of our participants was not the case in our sample. Interestingly, among those who voluntarily returned, pull factors were crucial.

Considerations concerning a re-emigration to the country of origin were dominated by economical motives. These results are relevant for two reasons. On one hand, they show that return in the study group was not sustainable; a point which will be discussed later. On the other hand, they also demonstrate that motives for migration change over time. When discussing the return of a refugee population, the same motives which initially lead to flight from the country of origin should not be assumed.

In this context, the duration of stay in Germany plays an important role. After up to 13 years in exile, it is inevitable that people have settled in and that a return to the home country marks yet another disruption in a refugee's biography. Of essential importance in Germany are therefore faster procedures in applying for asylum. This would provide clearer orientation for the refugees concerned.

During their time in exile, refugees accustom themselves not only to a mentality and cultural environment, but also to a standard of living. It is therefore not possible to shed light on the psychological question investigated in this study without considering the economical context. Ways out of this inequality regarding the standard of living of individuals are relatively complex, as they include considerations about the general repartition of goods and resources between countries. While the desire for a standard of living that meets the basic needs of an individual is defined as a human right by the United Nations, it is not recognized as a reason for asylum [62]. When analysing return from a psychological point of view, however, we cannot ignore the perspective of the returnee which also includes economic aspects.

This is also important from another point of view. In the interviews we found that mental health played a subordinate role in the entire return process, e.g. for the return agencies and the returnees themselves. Even though it was the focus of the study, participants focussed more strongly on economic factors. For this we would not approve the possible objection that returnees might be tending to state mental problems more strongly.

### Voluntariness and sustainability of return

The study demonstrated that different degrees of voluntariness can be identified. Approximately half of the VARP participants, who do actually return to their country of origin, return voluntarily (as opposed to those who are dropping out of the programme before the actual return). Those who returned voluntarily in our study were either elderly people and/or terminally ill and had the desire to die in their country of origin. The remaining participants reported returning with VARP in order to avoid deportation. Hence, refugees may be faced with the choice of returning voluntarily when asked to do so and perhaps gaining financial or other incentives (free mobility) as a result, or staying and risking forcible return at a later date [11]. That participants of VARP often do not return voluntarily can also be seen in the high drop-out rates in our study sample. Parallel to the return process, programme participants also initiated proceedings for a permanent stay in Germany and left the programme upon achieving their goal.

A further conclusion which can be drawn from study results is that the existence of VARPs does not increase the rate of voluntary returnees. While return assistance is thus seen as helpful once the decision to return has been made, VARPs do not facilitate the voluntary decision itself [8].

A further aspect which is often subject to discussion in this context is the sustainability of return. As cited above, UNMIK claims that „amongst key conditions for sustainability, returning migrants arguably need employment, housing, access to public and social services, education, public utilities, and security” [9,11]. If access to basic necessities is not available, the failings of reintegration can have 'ramifications for the wider society' [11]. As shown in our study, these basic needs are not met for the majority of participants. Many returnees live under or only slightly above the poverty line and have no employment. While the refugees examined in our study returned with assisted return programmes, a much larger number of people return without any support or are deported. At the same time, it must be considered that the conditions set by UNMIK are also not realised for large parts of the population who did not leave the country during the war period. Guaranteeing stable and prosperous living conditions for returnees in the country of origin would widen the gap between affected individuals and the rest of society and would probably increase conflicts which otherwise exist on a small scale. As such, it is therefore not only a question of how to make return sustainable, but how to make it sustainable on a community basis and not just for the individual returnee [11]. One possible solution may be the creation of programmes from which returnees and the local population who did not leave the country during the war benefit.

In his approach, Cassarino specifies factors which determine the success or failure of return [18]. The results of our study confirm this approach. As detailed above, Cassarino postulates the necessity of 'preparedness' which comprises the two elements 'willingness' and 'readiness'. In the study sample, 'willingness' was limited, with more than half of the sample claiming that their return was not voluntarily but rather to avoid forced return. According to Cassarino's definition, ‚readiness' was also not realised in the majority of study participants. In general, returnees did not have personal contacts and/or financial resources to prepare an existence after return. Since they were not allowed to work in Germany, they also were not able to develop professional skills or even lost them with time. Despite returning with state-sponsored programmes, the funds provided were start-up funds rather than a sustainable form of support. Only those participants with a network and the support of friends and families in the country of origin succeeded after return. Providing relief for this problem necessitates higher expenses for the individual returnee and a different approach including more integrated programmes with long-term assistance. While some German agencies involved in VARP promote such an approach, our study demonstrated that a consistent realisation is not pursued.

### Limitations

While every effort was made to include as many returnees as possible in our study, the attained sample size is relatively small. This lends the study the character of a pilot study. Further research with larger sample sizes should be undertaken in order to substantiate our findings. It is also recommended that the qualitative findings of our study be used as a basis for the generation of new variables which would allow a more detailed investigation of the research question.

The small sample size also limits the representativeness of findings. Unfortunately, a number of organisations involved in VARP in Germany did not refer clients to us. In cases of low feedback, organisations which did not refer clients were asked to explain why. Four organisations did not want to cooperate for fear that the data could be misused for political reasons, such as a campaign for or against the practice of VARP. Other organisations argued that the interview would be stressful for their clients. In some refugees, the fear of return was immense and organisations were not willing to refer them to the study.

Unfortunately there is very little information on the mental health of refugees in Germany which makes it difficult to estimate the representativeness of our results. This in fact stimulated us to perform this study. The only data which exists in Germany comes from an earlier study, which found a PTSD-prevalence rate of 40% among asylum seekers in Germany [19]. This is comparable to the PTSD-rates found in the present study. As far as we know there is no data available on demographic characteristics of refugees living in Germany. For returnees we also could not obtain general, i.e. nation-wide, information on demographic characteristics. Therefore we examined the demographic statistics of those organisations involved in VARP [41,63,64]. According to these statistics our sample is representative for returnees in Germany regarding age, gender, marital status, residence status and country of origin.

Given that the study includes returnees from a variety of organisations, in particular those which are more confident in their programmes, effects across all organisations may be even stronger and the current conclusions are therefore likely to be valid. It is also unfortunate that the situation in Iraq did not allow for follow-up interviews with some of the returnees.

Taking into consideration the general lack of information on refugees in Germany we limit our findings to the group of returnees in Germany who came as refugees and are returning now with assisted programs of voluntary return. Within that frame our findings are representative. In terms of voluntary return in Germany in general our study has the character of a pilot study. Further investigations with larger samples from different countries of origin should be performed to assure and deepen the results of the study presented in this paper.

## Conclusion

The current study shows that psychological strain among returnees participating in programmes of assisted voluntary return is already at a high level prior to return and even greater nine months after return. Most frequently diagnosed psychiatric disorders are affective disorders and PTSD.

As claimed by the UNHCR, "experience has shown that return itself is not enough....it needs to be 'successful' and 'sustainable'. Otherwise it could lead to renewed conflict and further displacement." [6]. An important prerequisite for successful and sustainable return is voluntariness. This was not realized in two thirds of the returnees included in our study. It was shown that monetary incentives and assistance programmes are not central in deciding whether or not to return, although participants who had decided to return did feel that assistance would help. In our study, participants who actually returned voluntarily were mostly elderly and terminally ill women. For future studies, we recommend separately analysing the data of those groups whose desire to return voluntarily is self-determined. It is likely that VARP objectives must be defined on a much smaller scale with specific target groups. Programmes should be given names which reflect their true nature, such as, for example, 'state-sponsored repatriation', in order not to generate unrealistic expectations in returnees. A further option would be to invest more heavily in VARPs and the reintegration of returnees.

Regarding content, programmes should focus not only on returnees but also on the population who did not leave the country during the war in order to avoid further problems in the country of origin.

## Abbreviations

AWO: Arbeiterwohlfahrt (Labour welfare); BAMF: Bundesamt für Migration und Flüchtlinge (Federal Office for Migration and Refugees); DSM: Diagnostic and Statistic Manual of Mental Disorders; EUROHIS: Health Interview Survey in Europe; ICD-10: International Classification of Diseases- 10^th ^edition; IOM: International Organisation for Migration; M.I.N.I.: Mini International Neuropsychiatric Interview; PDS: Posttraumtic Stress Diagnostic Scale; NGO: Non-Governmental Organisation; PTSD: Posttraumatic Stress Disorder; QoL: Quality of Life; SPSS: Statistical Package for the Social Sciences; UNHCR: United Nations High Commissioner for Refugees; UNMIK: United Nations Mission in Kosovo; VARP: Voluntary Assisted Return Program; WHO: World Health Organisation; WHOQOL-100 (questionnaire): World Health Organisation Quality of Life- 100 (items); WHOQOL-BREF (questionnaire): World Health Organisation: short form (French: bref) of the WHOQOL-100; ZIRF: Zentralstelle für Informationsvermittlung zur Rückkehrförderung (Central Information Office on Assisted Return)

## Competing interests

The authors declare that they have no competing interests.

## Authors' contributions

Concept: UvL, FN, TE; Data searching: UvL; Analysis: UvL; First draft: UvL; Critical revisions: UvL, TE; Final manuscript read and approved: UvL, TE, FN.

## Pre-publication history

The pre-publication history for this paper can be accessed here:


